# Metabolomic Characterization Reveals ILF2 and ILF3 Affected Metabolic Adaptions in Esophageal Squamous Cell Carcinoma

**DOI:** 10.3389/fmolb.2021.721990

**Published:** 2021-09-09

**Authors:** Bin Zang, Wen Wang, Yiqian Wang, Pengfei Li, Tian Xia, Xiaolong Liu, Di Chen, Hai-long Piao, Huan Qi, Yegang Ma

**Affiliations:** ^1^ Department of Thoracic Surgery, Cancer Hospital of China Medical University, Liaoning Cancer Hospital and Institute, Shenyang, China; ^2^ CAS Key Laboratory of Separation Science for Analytical Chemistry, Dalian Institute of Chemical Physics, Chinese Academy of Sciences, Dalian, China; ^3^ Department of Radiotherapy, The First Affiliated Hospital of Dalian Medical University, Dalian, China; ^4^ Department of Biochemistry and Molecular Biology, School of Life Sciences, China Medical University, Shenyang, China

**Keywords:** esophageal squamous cell carcinoma, metabolomics, interleukin enhancer binding factor 2, interleukin enhancer binding factor 3, acyl-carnitines, glycolysis

## Abstract

Esophageal cancer (EC) is a common malignant disease in eastern countries. However, a study of the metabolomic characteristics associated with other biological factors in esophageal squamous cell carcinoma (ESCC) is limited. Interleukin enhancer binding factor 2 (ILF2) and ILF3, double-stranded RNA-binding proteins, have been reported to contribute to the occurrence and development of various types of malignancy. Nevertheless, the underlying functions of ILF2 and ILF3 in ESCC metabolic reprogramming have never been reported. This study aimed to contribute to the metabolic characterization of ESCC and to investigate the metabolomic alterations associated with ILF2 and ILF3 in ESCC tissues. Here, we identified 112 differential metabolites, which were mainly enriched in phosphatidylcholine biosynthesis, fatty acid metabolism, and amino acid metabolism pathways, based on liquid chromatography–mass spectrometry and capillary electrophoresis–mass spectrometry approaches using ESCC tissues and paired para-cancer tissues from twenty-eight ESCC patients. In addition, *ILF2* and *ILF3* expression were significantly elevated in EC tissues compared to the histologically normal samples, and closely associated with PI3K/AKT and MAPK signaling pathways in ESCC. Moreover, in ESCC tissues with a high ILF2 expression, several short-chain acyl-carnitines (C3:0, C4:0, and C5:0) related to the BCAA metabolic pathway and long-chain acyl-carnitines (C14:0, C16:0, C16:0-OH, and C18:0) involved in the oxidation of fatty acids were obviously upregulated. Additionally, a series of intermediate metabolites involved in the glycolysis pathway, including G6P/F6P, F1,6BP, DHAP, G3P, and 2,3BPG, were remarkably downregulated in highly ILF3-expressed ESCC tissues compared with the corresponding para-cancer tissues. Overall, these findings may provide evidence for the roles of ILF2 and ILF3 during the process of ESCC metabolic alterations, and new insights into the development of early diagnosis and treatment for ESCC. Further investigation is needed to clarify the underlying mechanism of ILF2 and ILF3 on acyl-carnitines and the glycolysis pathway, respectively.

## Introduction

Esophageal cancer (EC) originating from the epithelium of the esophagus is the seventh most common malignancy and the sixth leading cause of death globally (Sung et al., 2021). There are two most common histologic subtypes of EC, esophageal squamous cell carcinoma (ESCC) and esophageal adenocarcinoma (EAC), of which ESCC comprises over 90% of EC cases (Bray et al., 2018). Although the morbidity and mortality of ESCC have recently shown a downward trend in China, the number of morbidities and deaths still accounts for more than half of the world (Chen et al., 2016). The therapeutic outcome of ESCC has been recently improved with multimodal treatments; however, the prognosis and survival of patients remain far from satisfactory, with 5-year survival rates of less than 20% (Merkow et al., 2014). It is believed that the diagnosis at the early stage and individualized precision treatment for ESCC would lead to a decrease in mortality. To achieve this, the biological characteristics of ESCC patients and robust biomarkers for the early diagnosis of ESCC are warranted to be identified.

Increasing evidence has shown that the progression of ESCC is closely related to the alterations in the biological activity of some metabolites (Abbassi-Ghadi et al., 2013; Huang et al., 2020). A high-throughput metabolomic analysis that focuses on endogenous low molecular weight metabolites can facilitate comprehensive identification of promising novel biomarkers as well as the discovery of novel therapeutic targets and preventive strategies (Wishart, 2016). Thus, comparative metabolomic profiling has been conducted for ESCC in various human biological samples, including urine, serum, and tissue, based on mainly nuclear magnetic resonance (NMR), gas chromatography–mass spectrometry (GC-MS), liquid chromatography–mass spectrometry (LC-MS), or capillary electrophoresis–mass spectrometry (CE-MS) (Jin et al., 2014; Zhang et al., 2017; Zhu et al., 2017; Tokunaga et al., 2018a). Although these studies identified a series of differential metabolites and suggested potential biomarkers for ESCC diagnosis, the results are still equivocal, and the associations between metabolomic characteristics and other biological factors of ESCC patients have rarely been explored and still remain unknown.

Interleukin enhancer binding factor 2 (ILF2, also known as NF45) and ILF3 (also known as NF90) function as a stable heterodimeric complex to stabilize mRNAs and regulate gene expression (Kiesler et al., 2010; Ye et al., 2017). The ILF2/ILF3 complex has been reported to participate in multiple cellular processes such as DNA repair and replication (Shamanna et al., 2011), mRNA stabilization (Jiang et al., 2015), transcription (Tsai et al., 2021), translation inhibition (Nourreddine et al., 2020), and micro-RNA biogenesis (Todaka et al., 2015; Higuchi et al., 2016). Recently, the role of ILF2 and ILF3 in tumors is emerging, and it has been found that they were involved in the occurrence and development of various types of malignancy, including gastric cancer (Yin et al., 2017; Liu et al., 2019), non–small-cell lung cancer (Ni T. et al., 2015; Cheng et al., 2018), breast cancer (Hu et al., 2013; Jin et al., 2018), and pancreatic carcinoma (Wan et al., 2015; Li et al., 2019). Additionally, ILF2 has been reported to be highly expressed in ESCC and suggested to be a prognostic factor for the poor outcome of ESCC patients (Ni S. et al., 2015; Wen-Jian et al., 2019). Mechanistically, ILF2 could promote ESCC cell growth and invasion by upregulating 14-3-3ε/Rac1/Tiam1 signaling (Wen-Jian et al., 2019), and ILF3 has been reported to participate in the process of IRF1 transcription activated by lncRNA IRF1-AS in ESCC (Huang et al., 2019). Nevertheless, the expression and significance of ILF3 in ESCC are still obscure, and the roles of ILF2 and ILF3 in ESCC metabolic reprogramming have never been reported.

Therefore, the aim of this study was to contribute to the metabolic characterization of ESCC and to investigate the associations between ILF2 as well as ILF3 and metabolomic alterations of ESCC tissues. To achieve this, using LC-MS–based and CE-MS–based metabolic profiling approaches, we analyzed the differences in metabolomic characteristics between ESCC tissues and paired para-cancer tissues from twenty-eight patients with ESCC. Meanwhile, our results from the analyses of publicly available clinical datasets demonstrated that both ILF2 and ILF3 were highly expressed in ESCC tissues compared with the noncancerous tissues and involved in multiple ESCC-related pathways and metabolic pathways. Furthermore, the respective expression of ILF2 and ILF3 in the abovementioned tissues as well as the associations between these two factors and metabolomic characteristics of ESCC tissues were analyzed. Our findings may provide evidence for the metabolic alterations regulated by ILF2 and ILF3 in the process of ESCC, and new insights into the development of early diagnosis and treatment for ESCC.

## Materials and Methods

### Subjects

Twenty-eight ESCC patients were recruited, and the paired tissue samples of ESCC tissues and para-cancer tissues were obtained between September 2019 and December 2019 from the Thoracic Surgery Department of the Cancer Hospital of China Medical University (Liaoning Cancer Hospital and Institute). All subjects signed a written informed consent form prior to the participation in this study. All the tissue samples were collected during surgery, and each of them was immediately stored in liquid nitrogen for 2 h and then stored at −80°C until analysis. This work was approved by the Ethics Committee of Cancer Hospital of China Medical University (Liaoning Cancer Hospital and Institute). The clinical and demographic features for study subjects and tumor characteristics are summarized in Table 1.

**TABLE 1:** Summary of clinical and demographic features for study subjects and tumor characteristics.

Characteristics	No. of subjects	Age (median, range), yr	Gender (n)		Tumor location (n)		
		**64.,52–76**	**Male**	**Female**	**Upper**	**Middle**	**Lower**
TNM stage							–
I/II	13	67,55–76	12	1	0	8	5
III	14	62,52–75	14	0	0	4	10
IV	1	62,62	1	0	0	1	0
Lymph metastasis							
Yes	15	62,52–75	15	0	0	5	10
No	13	67,55–76	12	1	0	8	5
ILF2/3 expression							
IFL2 high expression in EC tissues	13	64,52–75	13	0	0	5	8
IFL2 non-high expression in EC tissues	15	64,55–76	14	1	0	8	7
IFL3 high expression in EC tissues	16	63,52–75	16	0	0	6	10
IFL3 non-high expression in EC tissues	12	66,59–76	11	1	0	7	5

### Chemicals

High-performance liquid chromatography (HPLC)-grade chloroform, HPLC-grade methanol (MeOH), HPLC-grade acetonitrile (ACN), HPLC-grade 2-propanol (IPA), 98% formic acid, HPLC-grade ammonium acetate, and ultrapure water were used for solvent preparation and metabolites extraction. Internal standards in extraction solvent included FFA C16:0-d3, FFA C18:0-d3, carnitine C10:0-d3, carnitine C16:0-d3, and internal standards 1 (IS1) (Human Metabolome Technologies, H3304-1002).

### Metabolite Extraction

The extraction and analysis of metabolites were performed as previously described (Yan et al., 2019). In brief, fresh tissues were sheared and weighed when still frozen. Then, 600 µL ice-cold methanol with internal standards was added and subjected to a mixed grinding apparatus (Scientz-48) for homogenization (35 Hz, 2 min) followed by the addition of 600 µL chloroform and vortex for 30 s. After phase-breaking using 240 µL water and centrifugation (13,000 rpm, 4°C, and 15 min), the 420 µL hydrophilic layer was transferred for ultrafiltration through a 5-kDa cutoff filter, vacuum-dried, and stored at −80°C until CE-MS–based metabolite analysis. Next, the 420 µL hydrophobic layer was collected and freeze-dried for fatty acid analysis using LC-MS. Additionally, the 150 µL hydrophilic layer and 150 µL hydrophobic layer were freeze-dried for LC-MS–based carnitine and acyl-carnitines analysis.

### CE-MS Analysis and LC-MS Analysis

CE-MS analysis was conducted on CE (G7100A, Agilent) couple to time-of-flight (TOF) mass spectrometry (G6224A, Agilent). The fused silica capillary [50 µm i.d. × 80 cm, Human Metabolome Technologies (HMT), Tsuruoka, Japan] was used for sample separation, and the temperature of the capillary was controlled at 20°C. Detailed CE-MS methods were performed as previously described (Zeng et al., 2014). The qualitative analysis of metabolites was proceeded based on the pre-analyzed metabolite standard library (HMT), and peak extraction and identification were carried out with Quantitative Analysis software (Agilent).

Fatty acid analysis was performed using a Waters ACQUITYTM UHPLC system (Waters Corp, Milford, United States) coupled to AB SCIEX TripleTOF 5600 system (AB SCIEX, Framingham, United States). The C8 AQUITY column (2.1 mm × 100 mm × 1.7 µm, Waters, Milford, MA) was used for fatty acid separation. The mobile phases and gradient elution were used as previously described (Xuan et al., 2020). Development and validation of this rapid method was also described previously (Xuan et al., 2020). The MS parameters of a negative mode were as follows: ion spray voltage: 4500 V, curtain gas: 35 pound-force per square inch (psi), declustering potential (DP): −100 V, collision energy (CE): −10 V, and interface heater temperature: 550°C. Fatty acid identification was based on exact mass, retention time, and MS/MS pattern. Lipidview and Peakview workstations (AB SCIEX, United States) were used to check MS/MS information of fatty acids. Multiquant (AB SCIEX, United States) was used to obtain the area of fatty acids. The applied database search engines were HMDB (http://www.hmdb.ca/), Metlin (https://metlin.scripps.edu), and LIPID MAPS (http://www.lipidmaps.org/).

Carnitine and acyl-carnitines analyses were carried out on Agilent 6546 LC/Q-TOF. A C8 AQUITY column (2.1 mm × 50 mm × 1.7 µm, Waters, Milford, MA) was used for metabolite separation as described previously (Ouyang et al., 2018). The MS conditions in the positive mode were as follows: nebulizer pressure: 39 psi, dry gas temperature: 320°C, nitrogen flow: 8 L/min, capillary voltage: 4 kV, fragmentor: 150 V, and skimmer: 65 V. Metabolite identification was based on exact mass, retention time, MS/MS pattern, and available standard compounds (Yu et al., 2018).

### Data Processing and Statistical Analysis

The peak area of each metabolite was normalized according to the peak area of internal standard and tissue weight in a sample. Statistical significance between ESCC tissues and para-cancer tissues was evaluated by the Mann–Whitney *U* test, and *p* < 0.05 indicated significant. Partial least squares-discriminate analysis (PLS-DA) was performed using SIMCA software (Umetrics, Umea, Sweden, version 14.0), and heatmap cluster analysis of differential metabolites was performed by Multi Experiment Viewer software (MeV, version 4.8.1).

## Results

### Differential Metabolites Between ESCC and Paired Para-Cancer Tissues

**FIGURE 1:**
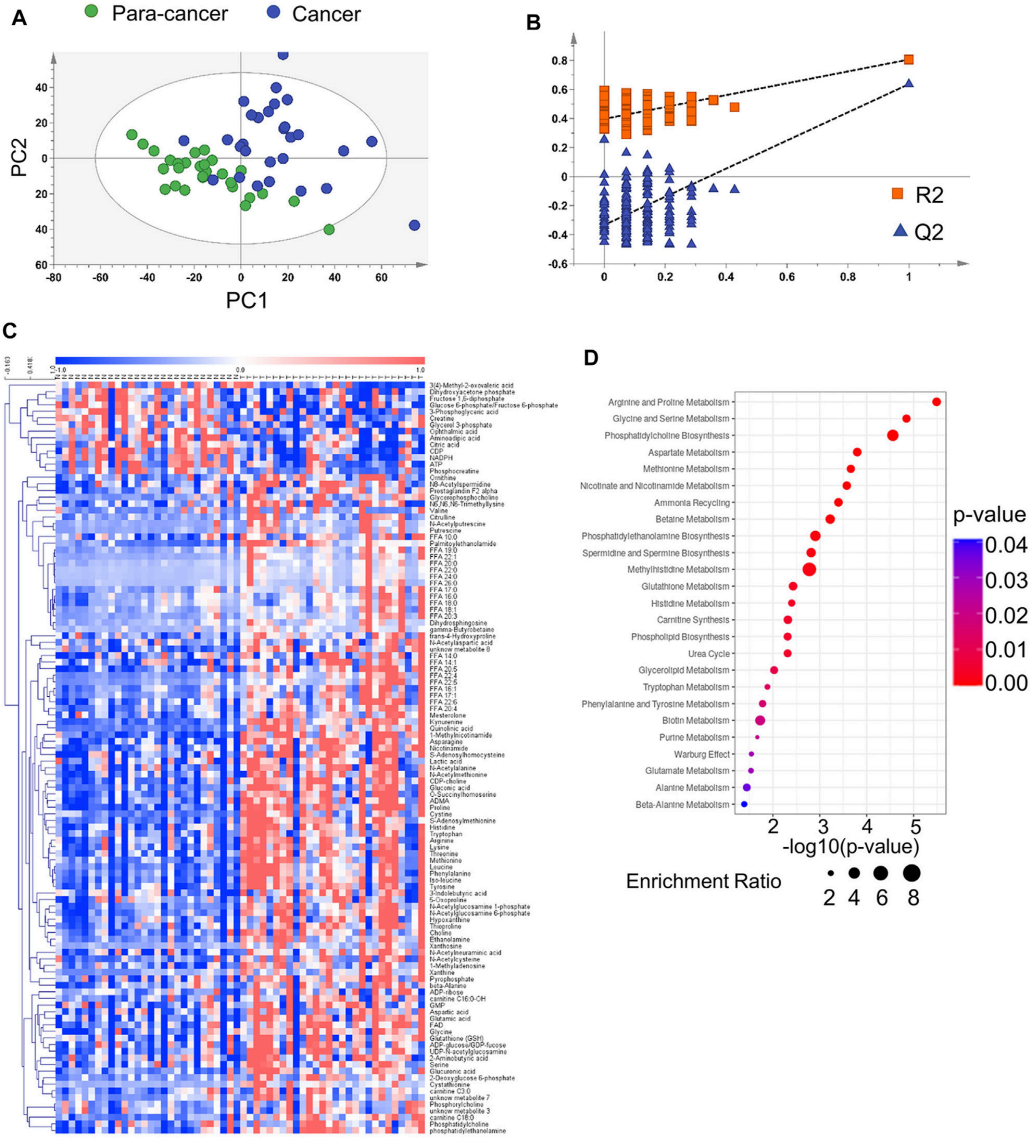
Metabolomic analysis of ESCC and para-cancer tissues. **(A)**. PLS-DA score plot of ESCC and para-cancer tissues. **(B)**. PLS-DA validation plot and para-cancer tissues. The Q2 and R2 indicated predictive ability and explanation ability of the model, respectively. The intercept of Q2 and R2 were 0.345 and 0.395, respectively. **(C)**. Heatmap analysis of all differential metabolites between and para-cancer tissues. **(D)**. MetaboAnalyst analysis of metabolic pathways by 112 differential metabolites (*p* < 0.05).

To contribute to the metabolic characterization of ESCC, we performed LC-MS–based and CE-MS–based untargeted metabolomic approaches using ESCC tissues and paired para-cancer tissues from twenty-eight ESCC patients. Based on the obtained metabolic profiling data, a PLS-DA model was established, and it showed an obvious separation between ESCC and para-cancer tissues on the score plots (Figure 1A). In addition, the model showed no overfitting after cross validation, and the intercepts of Q2 and R2 were −0.345 and 0.395, respectively (Figure 1B). In total, 112 differential metabolites between ESCC tissues and para-cancer tissues were identified with *p* < 0.05 by the univariate analysis, the relative levels of which were visualized on a plotted heatmap (Figure 1C). To determine the alterations in metabolic pathways relevant to ESCC, the pathway enrichment analysis was performed by using the online software MetaboAnalyst (www.metaboanalyst.ca) based on the 112 differential metabolites. The results showed that arginine and proline metabolism, glycine and serine metabolism, and phosphatidylcholine biosynthesis were the most obviously perturbed pathways in EC tissues (Figure 1D).

**FIGURE 2:**
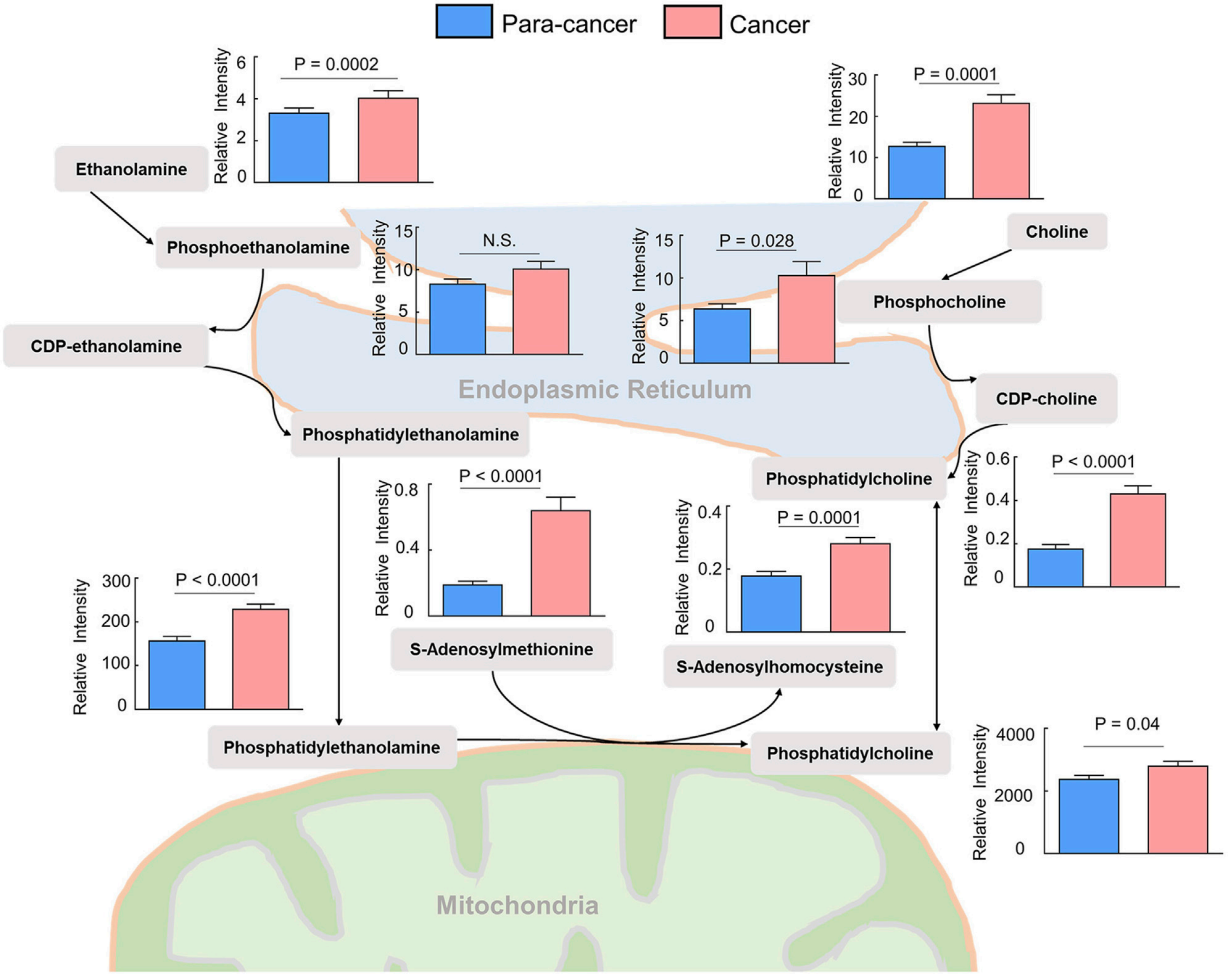
Upregulated phosphatidylcholine and phosphatidylethanolamine biosynthesis pathway in ESCC tissues compared with pericarcinous tissues. (*n* = 28. Data were expressed as mean ± SEM.)

**FIGURE 3:**
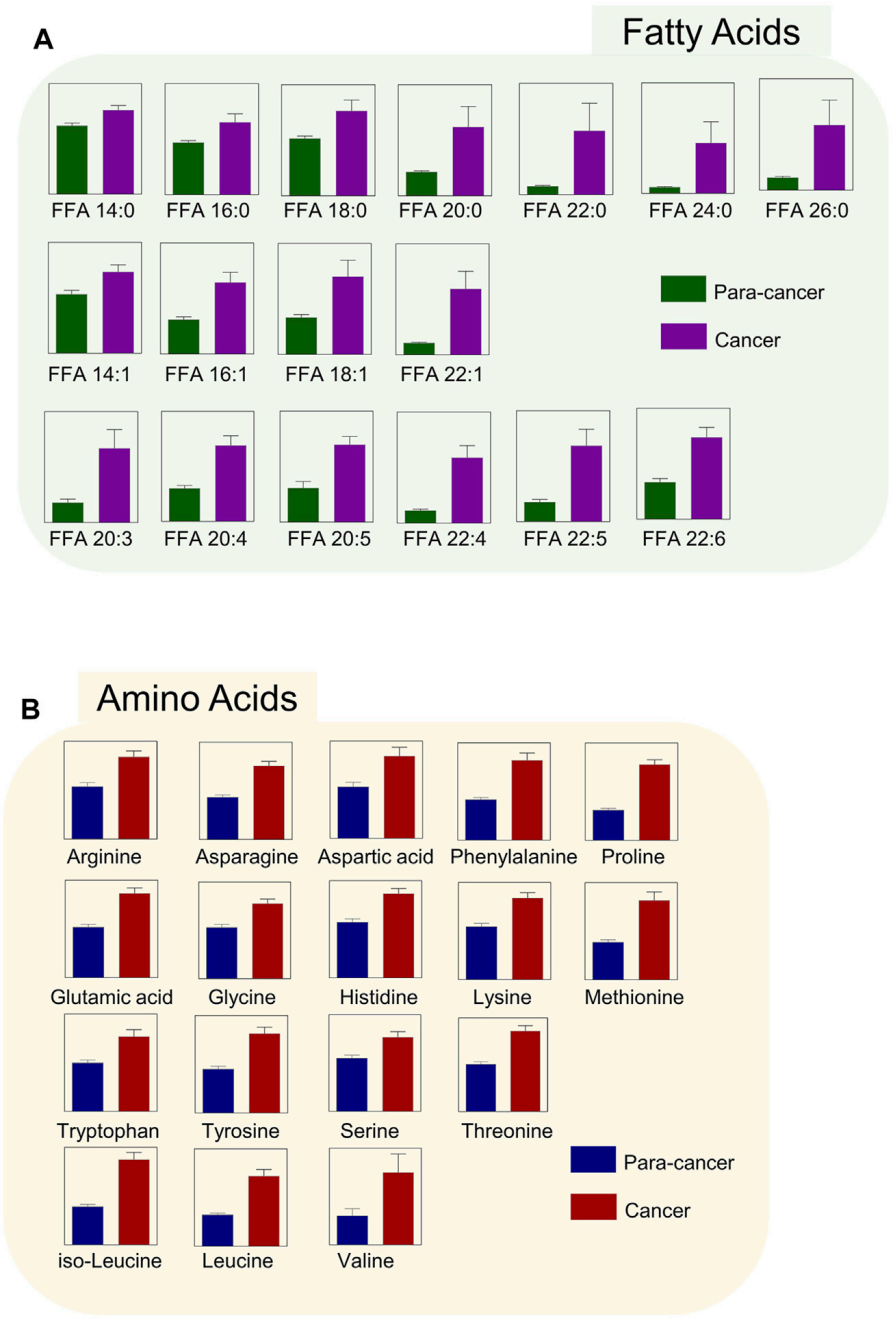
Upregulated **(A)** fatty acids and **(B)** amino acids were identified in ESCC tissues. (*n* = 28. Data were expressed as mean ± SEM, *p* < 0.05.)

Phosphatidylcholines (PC) and phosphatidylethanolamine (PE) are the two main phospholipid components which are essential for the structure of biological membranes and signal transduction of cells (Cheng et al., 2016). Several cancer metabolomic studies have demonstrated that the levels of choline and its metabolites were significantly dysregulated in cancer prognosis (Iorio et al., 2010; Rysman et al., 2010). Here, we characterized the differential metabolites related to the synthetic pathways of PC and PE, and found that almost every intermediate metabolite in these pathways was upregulated in ESCC tissues compared to the para-cancer tissues (Figure 2). Additionally, based on fold ≥ 1.2 and *p* < 0.05 between ESCC tissues and the para-cancer tissues, 17 free fatty acids (FFAs), including saturated and unsaturated fatty acids, showed obvious upregulation in ESCC tissues (Figure 3A). Moreover, as shown in Figure 3B, amino acids presented fold ≥ 1.2 and *p* < 0.05 between ESCC tissues and the para-cancer tissues, which were elevated in ESCC tissues. Particularly, the branched-chain amino acids (BCAAs), including leucine, isoleucine, and valine, exhibited a significant increase in ESCC tissues (Figure 3B). These findings suggested a dysregulation of lipid metabolism and amino acid metabolism in ESCC tissues.

### The Related Biological Pathways of ILF2 and ILF3 in ESCC

**FIGURE 4:**
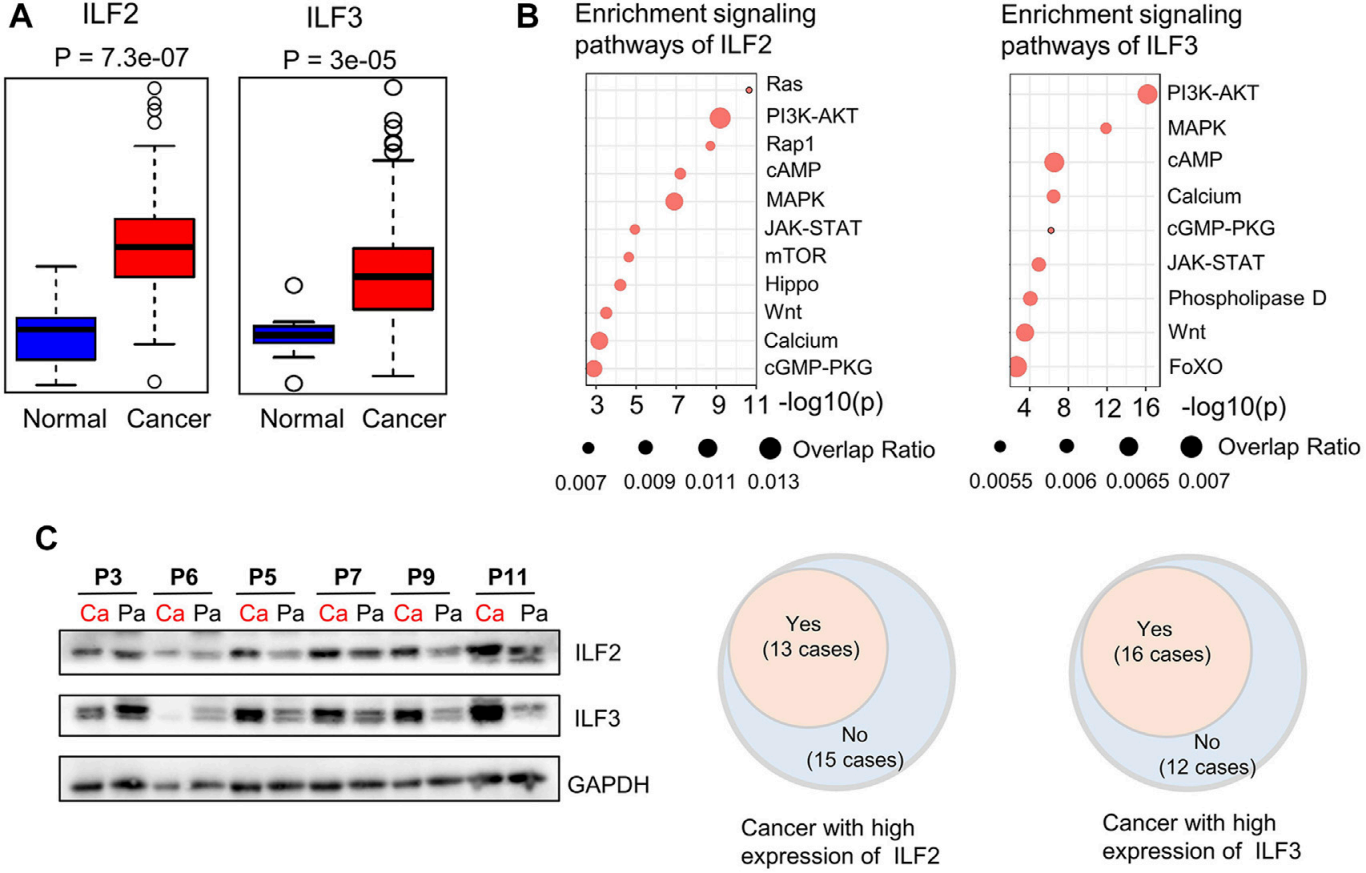
Bioinformatic analysis of ILF2 and ILF3 related with ESCC and Western blot analysis of ILF2 and ILF3 in 28 cases. **(A)**. Gene-expression analysis of ILF2 and ILF3 in EC according to public databases [n (cancer) = 182; n (normal) = 286]. **(B)**. Prediction of signaling pathways possibly affected by ILF2 and ILF3 in ESCC. **(C)**. Western blot analysis of ILF2 and ILF3 expression in 28 ESCC cases. GAPDH was used as a loading control in shown Western blot strips of representative 6 cases. (Ca indicated cancer tissues; Pa indicated para-cancer tissues.)

Although previous studies have demonstrated that ILF2 is involved in the progression of ESCC (Ni S. et al., 2015; Wen-Jian et al., 2019), the precise role and regulatory mechanisms of ILF2 and ILF3 in ESCC remain undefined. Here, we first performed a statistical analysis of the gene expression of *ILF2* and *ILF3* in EC based on the dataset of EC from the Cancer Genome Atlas (TCGA). As shown in Figure 4A, the elevated *ILF2* and *ILF3* expression were observed in EC samples (*n* = 184) compared with histologically normal samples (*n* = 11). Commonly, such abnormally expressed genes in cancer are closely related to the biological regulation of the development of EC. Hence, the signal pathways, that is, ILF2 and ILF3, that may participate in modulation during ESCC were predicted based on the TCGA data and KEGG pathway. The results from the Fisher exact test showed that both ILF2 and ILF3 were closely correlated with the phosphoinositol-3-kinase (PI3K)/AKT signaling pathway and mitogen-activated protein kinase (MAPK) signaling pathway, which are the downstream of the Ras signaling pathway (Figure 4B). Of note, the previous study integrated all single nucleotide variants and copy number alterations from the 158 ESCC cases, and identified genomic alterations of several critical signaling pathways, which included RTK-Ras and AKT pathways (Song et al., 2014). Additionally, such results were also supported by another study based on the genomic analysis of ESCC, which revealed that the PI3K-AKT signaling pathway is critical for the development and progression of ESCC (Chang et al., 2017). Therefore, we speculate that ILF2 and ILF3 may play an important role in the progression and prognosis of ESCC. Furthermore, we performed the gene set enrichment analysis (GSEA) to investigate possible signaling pathways and mechanisms through which ILF2 and ILF3 functioned to regulate ESCC. On the basis of FDR <0.05 and the normalized enrichment score, the obviously enriched biological pathways are shown in Supplementary Figure S1 and S2. We found that ILF2 was in close connection with Ras and Rap1 signaling pathways in ESCC, which was consistent with the results from the Fisher exact test. In addition, several metabolic pathways were also shown to be remarkably associated with ILF2 and ILF3, suggesting a potential biological function of these two factors in regulating metabolic reprograming of ESCC.

### Association Between ILF2 and Metabolic Characteristics in ESCC

In order to explore the regulatory mechanisms that ILF2 and ILF3 may participate in the metabolic disorders of ESCC tissues, we examined the expressed levels of these two proteins in ESCC tissues and paired para-cancer tissues. It was found that 13 of 28 ESCC patients exhibited high ILF2 expression in ESCC tissues compared to the paired para-cancer tissues (Figures 4C,Supplementary Figure S3). According to the expression levels of ILF2 in ESCC tissues, the 28 patients were divided into two groups: the patients with a higher expression of ILF2 in cancer tissues than the para-cancer tissues were grouped together (*n* = 13, group Ⅱ) and others were another group (*n* = 15, group Ⅰ).

**FIGURE 5:**
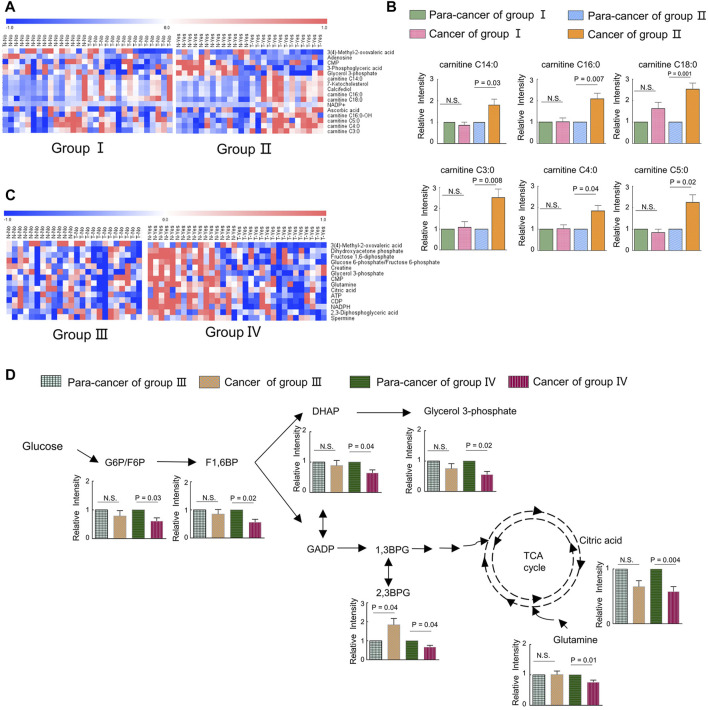
Metabolic adaptions related with a highly expressed ILF2 and ILF3 in ESCC. **(A)**. Heatmap analysis of differential metabolites according to the expression of ILF2 in ESCC tissues. Patients in group Ⅱ exhibited higher ILF2 levels in tumor tissues than those in the para-tumor tissues, and other cases were in group Ⅰ. **(B)**. Alterations of acyl-carnitines in ESCC cases with (*n* = 13, group Ⅱ) or without (*n* = 15, group Ⅰ) highly expressed ILF2 in tumor tissues. **(C)**. Heatmap analysis of differential metabolites according to the expression of ILF3 in ESCC tissues. Patients in group Ⅳ exhibited higher ILF3 levels in tumor tissues than those in the para-tumor tissues and others were in group Ⅲ. **(D)**. Influenced glycolysis pathway in ESCC cases with (*n* = 16, group Ⅳ) or without (*n* = 12, group Ⅲ) highly expressed ILF3 in tumor tissues. Data were expressed as mean ± SEM.

To uncover the alterations in metabolic pathways specifically associated with ILF2 protein levels, we analyzed the metabolic differences between the ESCC tissues and para-cancer tissues of patients in each group. Following excluding the metabolites with the same changes between the two groups, a total of 16 metabolites with different trends were identified, which were 3-methyl-2-oxovaleric acid, adenosine, CMP, 3-phosphoglyceric acid, glycerol 3-phosphate, 7-ketocholesterol, calcifediol, NADP+, ascorbic acid, carnitine C14:0, carnitine C16:0, carnitine C16:0-OH, carnitine C18:0, carnitine C3:0, carnitine C4:0, and carnitine C5:0 (Figure 5A). Among these metabolites, we found that in ESCC tissues with high ILF2 expression, 3-methyl-2-oxovaleric acid, the abnormal degradation product of BCAA was significantly downregulated, and several short-chain acyl-carnitines (C3:0, C4:0, and C5:0) related to the BCAA metabolic pathway were obviously upregulated (Figure 5B). In addition, four long-chain acyl-carnitines (C14:0, C16:0, C16:0-OH, and C18:0) which were involved in the oxidation of fatty acids were remarkably increased in ESCC tissues with high ILF2 expression (Figure 5B). Nevertheless, the levels of these metabolites in ESCC tissues with non-high expression of ILF2 showed no significant difference compared to the para-cancer tissues (Figure 5B). These findings suggested that ILF2 may play a critical role in the abnormal metabolic procession of BCAAs and fatty acids in ESCC.

### Association Between ILF3 and Metabolic Characteristics in ESCC

We also investigate the possible metabolic pathways specifically related to ILF3 protein levels in ESCC. Similarly, 28 ESCC patients were categorized into two groups based on the protein expression of ILF3: the one group (Group Ⅳ) included 16 patients with a higher ILF3 expression in tumor tissues than the para-tumor tissues; the other group (Group Ⅲ) consisted of the remaining 12 patients (Supplementary Figure S1). The metabolic differences between the ESCC tissues and para-cancer tissues of patients within each group were also analyzed. After excluding the metabolites with the same alterations between the two groups, a total of 14 characteristic metabolites with different changing trends were identified, including 3-methyl-2-oxovaleric acid, dihydroxyacetone phosphate (DHAP), fructose 1,6-diphosphate (F1,6BP), glucose 6-phosphate/fructose six- phosphate (G6P/F6P), creatine, glycerol 3-phosphate (G3P), CMP, glutamine, citric acid, ATP, CDP, NADPH, 2,3-diphosphoglyceric acid (2,3BPG), and spermine (Figure 5C). Of note, 3-methyl-2-oxovaleric acid, which was downregulated in ESCC tissues with high ILF2 expression, was also significantly decreased in highly ILF3-expressed ESCC tissues in comparison with the corresponding para-cancer tissues, suggesting that there may be a close relationship between these two binding factors and the metabolism of BCAAs during the progression of ESCC. Additionally, several intermediate metabolites involved in the glycolysis pathway, such as G6P/F6P, F1,6BP, DHAP, G3P, and 2,3BPG, were remarkably downregulated in ESCC tissues with high ILF3 expression (Figure 5D). Meanwhile, citric acid involved in TCA cycle and glutamine were also obviously decreased in highly ILF3-expressed ESCC tissues compared with the corresponding para-cancer tissues (Figure 5D). It is well known that glycolysis, TCA cycle, and glutamine mechanism are important energy metabolism pathways. Therefore, our findings indicated that ILF3 protein may exhibit a close connection with the energy metabolism during the progression of ESCC.

## Discussion

In the present study, we employed LC-MS–based and CE-MS–based metabolomics as well as bioinformatics to contribute to the metabolic characterization for ESCC and to explore the relationship of ILF2 and ILF3 with the metabolomic characteristics in ESCC tissues. First, we compared the metabolomes of ESCC tissues with paired para-cancer tissues from twenty-eight patients with ESCC, and identified 112 differential metabolites involved in various metabolic pathways, mainly including but not limited to amino acid metabolism, phosphatidylcholine biosynthesis, and fatty acid metabolism. Then, bioinformatic analysis showed that both ILF2 and ILF3 were highly expressed in ESCC tissues, and predicted the related signaling pathways and metabolic pathways of these two factors in ESCC tissues. Furthermore, according to the expression levels of ILF2 and ILF3 in EC tissues, our findings revealed that highly expressed ILF2 and ILF3 could affect acyl-carnitines and the glycolysis pathway, respectively.

Accumulating evidence indicates dysregulation of choline metabolism that is characterized by increased choline-containing compounds in various cancers (Glunde et al., 2015; Xiong et al., 2015). A significant increase of phosphocholine levels in breast and ovarian cancer has been reported (Iorio et al., 2005; Eliyahu et al., 2007). PCs were identified as a main class of perturbed metabolites in EC serum samples (Mir et al., 2015). Several previous studies found that choline was obviously increased in ESCC tissue (Zhang et al., 2017; Liang et al., 2019). Consistent with these findings, we also observed elevated choline levels in ESCC tissues compared with the para-cancer tissues. Moreover, the levels of downstream metabolites of choline, including phosphocholine, CDP-choline, and PC, were all significantly increased in ESCC tissues, indicating the upregulated choline metabolism in ESCC patients. In addition to the choline metabolism, the metabolism of ethanolamine, a critical fatty acid for cellular membranes, also plays an important role in lipid synthesis. Here, ethanolamine and its metabolites, including phosphoethanolamine, CDP-ethanolamine, and PE, were all identified to be increased in ESCC tissues. It has been reported that PC and PE are significantly increased in cancer cells and solid tumors, and are in close connection with functions such as membrane-mediated cellular signaling and energy storage (Ackerstaff et al., 2003). Hence, dysregulation of choline and ethanolamine metabolism in this study suggested aberrant metabolic characterization and cellular signaling in ESCC.

Fatty acids (FAs) are critical endogenous molecules for cellular energy storage, membrane formation, and signal transmission. It has been reported that upregulation of fatty acid synthesis is one of the most aberrant metabolic features of tumors and is required for membrane proliferation, energy consumption, lipid droplet formation, and signaling generation in various cancers (Chen and Li, 2017). The ion intensities of several FAs were found to be an increasing trend from the muscle to the epithelium to cancer tissue in ESCC, which was in good agreement with the expression of FASN, a critical metabolic enzyme for the *de novo* synthesis of FAs (Sun et al., 2019). The previous study reported that a series of FAs, such as stearic acid, oleic acid, aminomalonic acid, and linoleic acid, were higher in the tumor tissues of ESCC patients than in the normal tumor-adjacent tissues (Zhang et al., 2017). Consistent with these findings, our study also observed a significant increase of various FAs in ESCC tissues, including saturated and unsaturated FAs. Therefore, elevated FA levels may serve as potential biomarkers for ESCC diagnosis, and targeting FA biosynthesis may provide potent therapeutic strategies as well as promising anticancer drugs for ESCC treatment.

In addition to FA biosynthesis, the β-oxidation of FAs that release large quantities of ATP and generates reactive oxygen species also plays an important role in the progression of cancer (Wang et al., 2019). Carnitines and acyl-carnitines, the critical intermediates in the process of FA β-oxidation, are responsible for the transport of long-chain FAs across the mitochondrial membranes for energy generation and short-chain FAs from the mitochondria into the cytosol (Borum, 1996). Compared with the para-cancer tissues, ESCC tissues exhibited significantly higher levels of long-chain acyl-carnitines C16:0-OH and C18:0, reflecting the dysregulation of the β-oxidation of long-chain FAs in ESCC tissues. Interestingly, the present results showed that several long-chain acyl-carnitines (carnitines C14:0, C16:0, C16:0-OH, and C18:0) were obviously increased in ESCC tissues with a high ILF2 expression compared to the corresponding para-cancer tissues, whereas the levels of these metabolites showed no significant difference between ESCC tissues with a non-high expression of ILF2 and the para-cancer tissues. It has been reported that ILF2 can maintain mitochondrial homeostasis, especially oxidative phosphorylation, and promote the progression of small lung cancer *via* interacting with E2F1, which is a key transcriptional activator involved in the modulation of mitochondrial functions (Zhao et al., 2019). Notably, recent studies have indicated that E2F1 and E2F2 regulate the expression of CPT2, an important enzyme for FA β-oxidation, and establish a lipid-rich environment for hepatocarcinogensis (Gonzalez-Romero et al., 2021). Combined with these findings, we proposed that ILF2 might exhibit a close relationship with FA β-oxidation in ESCC. However, the underlying regulatory mechanism of ILF2 involved in the β-oxidation of long-chain FAs remains to be further explored.

In this study, the levels of most amino acids, including essential and nonessential amino acids, in ESCC tissues were significantly higher than those in nontumor counterparts, which was in line with the results from the previous studies (Tokunaga et al., 2018b). We speculated that such accumulation of almost overall amino acids in ESCC tissues was presumably attributed to the hyperactive autophagic protein degradation or amino acid transporters (Kobayashi et al., 2005; Morselli et al., 2009). Among these upregulated amino acids, BCAAs, including valine, leucine, and isoleucine, are a class of indispensable amino acids, and serve as critical nutrient sources in the synthesis of body proteins (Sivanand and Vander Heiden, 2020). It has been reported that BCAA metabolism is closely associated with other metabolic pathways, such as TCA cycle, oxidative phosphorylation, *de novo* nucleotide, and amino acid synthesis, and can satisfy several requirements that are critical for tumor cell proliferation (Sivanand and Vander Heiden, 2020). Thus, the dysregulated BCAA metabolism is widely prevalent in multiple cancer types and often serves as a marker of disease pathology.

One interesting finding from our study was that the levels of three short-chain acyl-carnitines (C3:0, C4:0, and C5:0), by-products of BCAA catabolism, were all obviously increased in ESCC tissues with high ILF2 expression. C3 acyl-carnitine is a by-product of both isoleucine and valine catabolism, reflecting the propionyl CoA pool (Newgard, 2012). C4 acyl-carnitine includes two isotypes, butyrylcarnitine and isobutyrylcarnitine, which are generated from fatty acid oxidation and valine catabolism, respectively (Chen et al., 2020). C5 acyl-carnitines comprised isovalerylcarnitine and α-methylbutyryl species, which are the intermediates in mitochondrial leucine and isoleucine catabolism, respectively (Newgard, 2012). Such accumulation of C3, C4, and C5 acyl-carnitines in ILF2-high expressed ESCC tissues suggested that ILF2 might be a close connection with dysregulated BACC catabolism. Moreover, 3-methyl-2-oxovaleric acid, a monocarboxylic acid derived from the catabolism of isoleucine, was obviously decreased in both ILF2 and ILF3-high expressed ESCC tissues. It has been reported that 3-methyl-2-oxovaleric acid can induce mitochondrial oxidative energy metabolism in skeletal myocytes *via* the cAMP-PKA-p38 MAPK pathway (Whitehead et al., 2021). A rate-limiting enzyme in BCAA catabolism, named BCKDK, has also been reported to promote colorectal cancer tumorigenesis *via* upregulating the MAPK pathway by phosphorylating MEK at Ser221 (Xue et al., 2017). Consistently, the data of our bioinformatics analysis indicated that both ILF2 and ILF3 were closely correlated with the MAPK signaling pathway in ESCC. Hence, these findings suggested that the abnormal BCAA metabolism in ESCC might be driven in part by the ILF2/ILF3 complex, but the mechanism by whether involvement of the MAPK signaling pathway remains to be explored.

Accumulating evidence has shown that ILF3 is abnormally expressed in a series of malignancies, and is involved in tumor proliferation, metastasis, and invasion (Hu et al., 2013; Jiang et al., 2015; Liu et al., 2019). However, the action and consequence of ILF3 in ESCC are not well elucidated. The present study found that ILF3 expression was obviously elevated in ESCC samples compared to the histologically normal samples, which suggested that ILF3 might be one of the factors promoting the carcinogenesis and development of ESCC. ILF3 has been reported to function as a critical regulator for the serine-glycine one-carbon (SGOC) pathway in colorectal cancer (CRC) *via* EGFR-ERK axis followed by E3 ligase speckle-type POZ protein (SPOP)-mediated poly-ubiquitination and degradation of ILF3 (Li et al., 2020). Moreover, the above studies demonstrated that the activity of intracellular lactate dehydrogenase and extracellular acidification rate was decreased in ILF3-knockdown CRC cells (Li et al., 2020). Here, we showed that several key intermediate metabolites involved in the glycolysis pathway were significantly decreased in ESCC tissues with high ILF3 expression compared with the corresponding para-cancer tissues. It is worthwhile to point out that there is a complex network between SGOC and glycolysis pathways, and the glycolytic intermediates are important for SGOC (Kottakis et al., 2016). Taken together, these findings suggested that ILF3 might play a critical role in the cross talk between glycolysis and SGOC pathways.

## Conclusion

In the current study, we identified the dysregulation of phosphatidylcholine biosynthesis, fatty acid metabolism, and amino acid metabolism in ESCC tissues using LC-MS–based and CE-MS–based metabolomic approaches. Moreover, our results from bioinformatics analysis indicated that elevated *ILF2* and *ILF3* expression in ESCC were closely associated with PI3K/AKT and MAPK signaling pathways. Furthermore, a series of acyl-carnitines, including short-chain acyl-carnitines and long-chain acyl-carnitines, were found to be positively correlated with the levels of ILF2 in ESCC tissues. Our data additionally demonstrated that elevated ILF3 expression in ESCC tissues was negatively correlated with the levels of several key intermediate metabolites in the glycolysis pathway. In the future, the regulatory mechanism of ILF2 and ILF3 on acyl-carnitines and glycolysis pathway, respectively, should be further explored.

## Data Availability

All datasets generated for this study are included in the article/Supplementary Material. The metabolomics data presented in the study are deposited in the EMBL-EBI MetaboLights repository, with the accession number MTBLS3333 (Haug et al., 2020).
