# Supplementary material for: Metabolomic Characterization Reveals ILF2 and ILF3 Affected Metabolic Adaptions in Esophageal Squamous Cell Carcinoma
Source: Front Mol Biosci. 2021 Sep 9;8:721990. doi: 10.3389/fmolb.2021.721990 (PMC8459612; doi:10.3389/fmolb.2021.721990)
Supplement: Supplementary file 1 [file DataSheet1_v1.DOCX]

**Supplementary**

**Metabolomic characterization reveals ILF2 and ILF3 affected metabolic adaptions in esophageal squamous cell carcinoma**


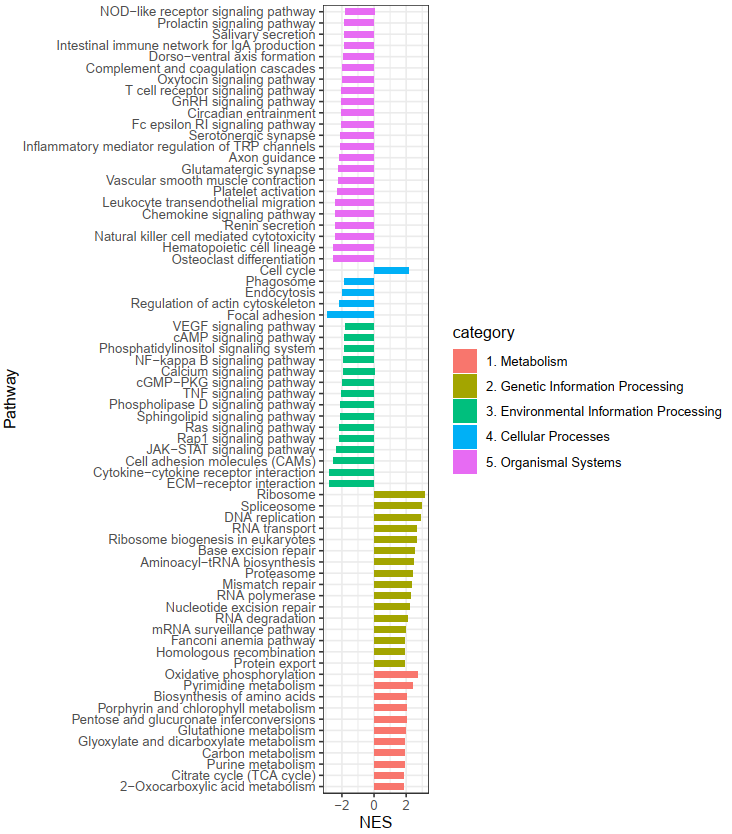


# Figure S1. Prediction of signaling pathways possibly affected by *ILF2* in ESCC based on pathways in KEGG.

#
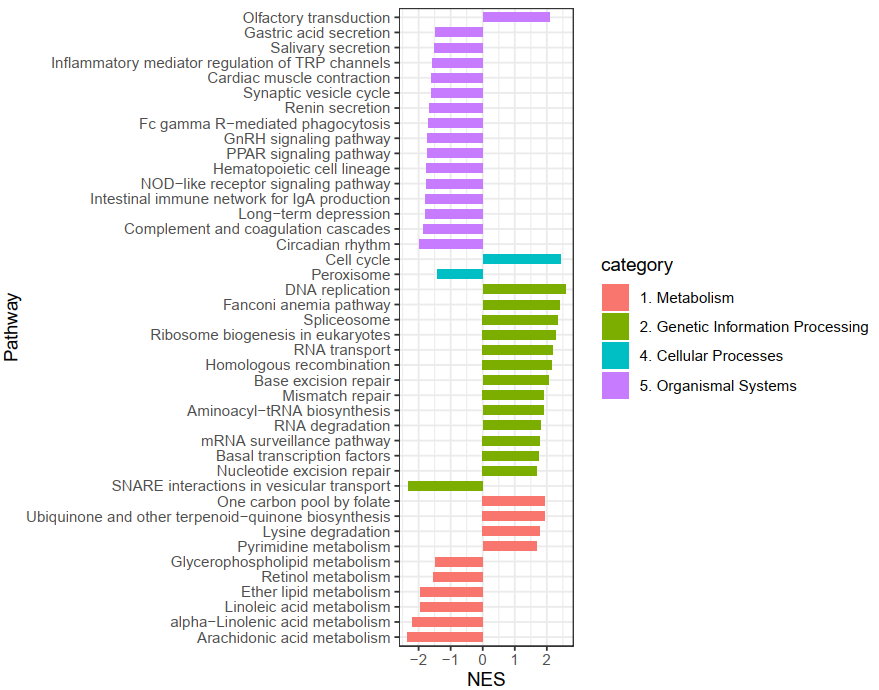


# Figure S2. Prediction of signaling pathways possibly affected by *ILF3* in ESCC based on pathways in KEGG.

**
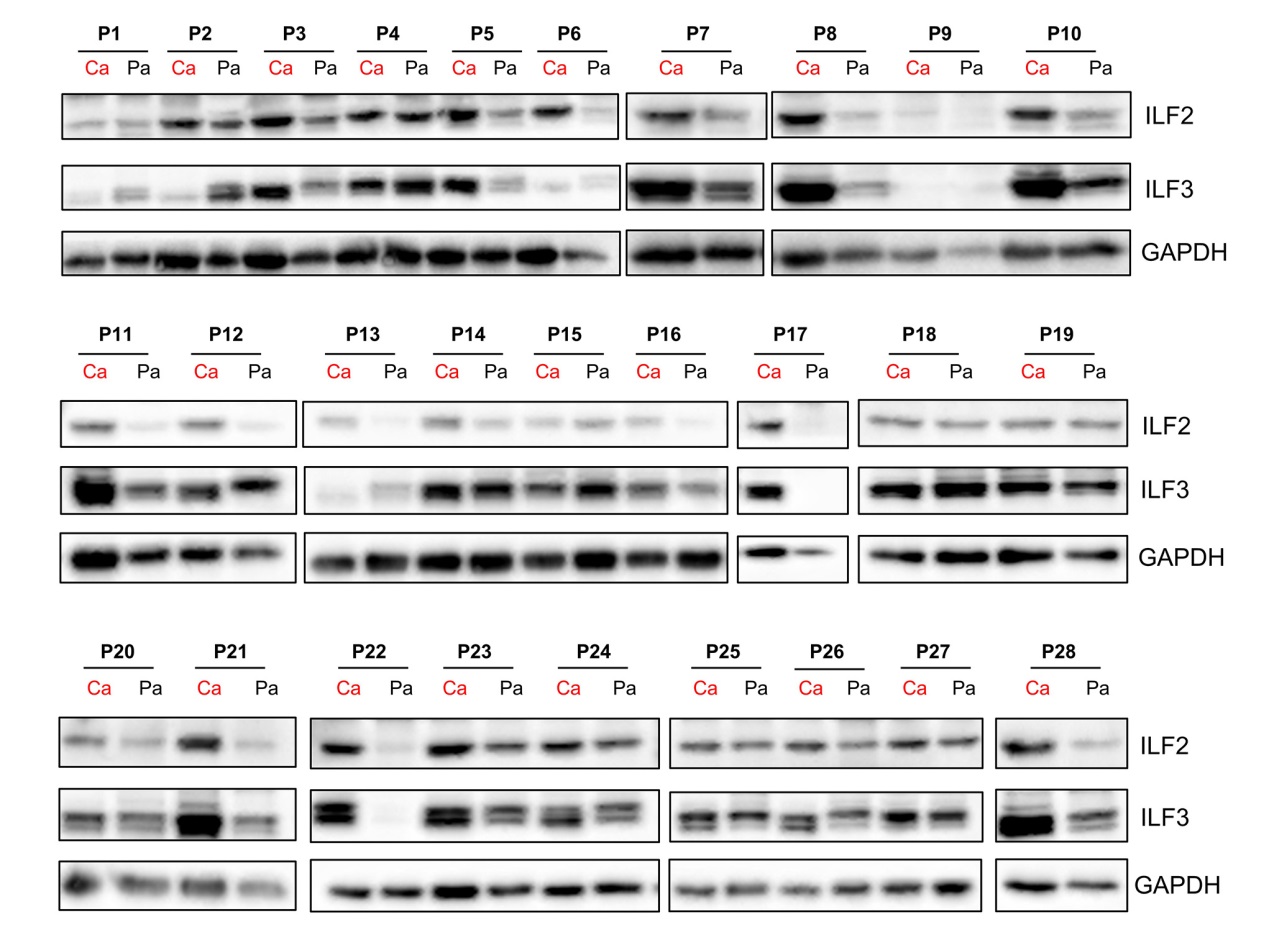
**

# Figure S3. Western blot analysis of ILF2 and ILF3 in 28 paired ESCC tissues. GAPDH was used as a loading control. Ca indicated cancer tissues; Pa indicated para-cancer tissues.
